# Complete mitochondrial genome analysis of *Lingula anatina* from Korea (Brachiopoda, Lingulida, Lingulidae)

**DOI:** 10.1080/23802359.2017.1407711

**Published:** 2017-11-24

**Authors:** Mustafa Zafer Karagozlu, Seong-Geun Kim, Do Dhin Thinh, Chang-Bae Kim

**Affiliations:** Department of Biotechnology, Sangmyung University, Seoul, Korea

**Keywords:** Brachiopoda, Lingulida, Lingulidae, complete mitogenome, *Lingula anatina*

## Abstract

In this study, complete mitochondrial genome of the *Lingula anatina* (Lamark, 1801) from Korea has been sequenced and analysed, and compared with previous complete mitochondrial genome record from Japan. The mitogenome is 25,790 bp and composed of 13 protein-coding genes, two ribosomal RNA and 34 tRNA. In comparison with previous record, there are dramatically changes in structure between two records. Additionally, phylogenetic tree of *L. anatina* in Brachiopoda reconstructed due to 12 protein-coding genes of mitochondrial genome. The results showed that the Korean *L. anatina* positioned in Brachiopoda and the closest species is the *L. anatina* from the Japan. This study provides the second complete mitochondrial genome for the species.

*Lingula anatina* is one of the most primitive species in brachiopods and they also approved as living fossil (Yang et al. 2013). DNA barcoding researches due to on cytochrome c oxidase subunit I (COI) and elongation factor 1 alpha (EF-1α) genes from specimens collected from different localities in this species also showed variations on lengths of gene and amino acid sequences (Reyment et al. 2007; Yang et al. 2013). Previously, it has been declared that the complete mitochondrial genome structure of *L anatina* which collected from Japan has unusual characteristics on genome size, gene order and elongated genes (Endo et al. 2005). In this study, complete mitochondrial genome of *L. anatina* from Korea sequenced, analysed and compared with previous record. This is the second complete mitochondrial genome record for the species.

The specimen was collected on February, 2016 from western coastal area of South Korea, Incheon 37°26'53″N, 126°22'13″E. The specimens were identified by DNA barcoding (Kim et al. 2017) and deposited in Department of Biotechnology, Sangmyung University, Korea University (SMBR0001) with 97% ethanol preservation. The mitochondrial DNA was extracted from whole body of the specimens. Methods for complete mitochondrial genome sequencing and phylogenetic tree reconstruction described previously (Karagozlu et al. 2016).

The size of the mitogenome is 25,790 bp (GenBank accession no. KX774482). There is a complete mitochondrial genome of *L. anatina* recorded from Japan (AB178773). It is approximately 3000 bp longer than Korean *L. anatina* from non-coding area (Endo et al. 2005). The mitochondrial genome is composed of 13 protein-coding genes, 2 ribosomal RNA and 34 tRNA. In Japanese record, it has 14 protein-coding genes (*Atp8* replicated), two ribosomal RNAs and 27 tRNA. Same as previous record, the all genes encoded on the majority strand, there is no any gene that encoded on the minority strand. The nucleotide composition of the genome is 26.2% A, 15.8% C, 21.1% G and 36.9% T. Total A–T content is 63.2% for complete mitochondrial genome. There are 34 tRNA which lengths are between 64 bp and 72 bp. In the mitogenome tRNA- Glu, tRNA-Leu, tRNA-Met, tRNA-Ser, tRNA-Trp repeated two times while tRNA-Gly repeated 10 times.

Additionally, the phylogenetic tree of the phylum Brachiapoda due to 12 protein-coding genes of mitochondrial genome (excluding ATP8) reconstructed to investigate phylogenetic relationships of *L. anatina* (Figure 1). Due to results *L. anatina* is positioned in the phylum Brachiopoda. The closest species to Korean *L. anatina* is Japanese *L. anatina*. The clade consists of *L. anatina* which represents the subphylum Linguliformea is earlier diverged than the subphyla Phoroniformea and Rhynchonelliformea in the Brachiapoda. The previously nuclear small subunit rRNA gene (Cohen et al. 1998) and e cytochrome c oxidase subunit I (COI) gene (Saito et al. 2000) based studies also support the mitochondrial protein coding gene-based phylogeny. To investigate evolution of *L. anatina*, number of complete mitochondrial genome data from different localities should increase. This study provides additional data for the Brachiopoda phylogeny.

**Figure 1.**
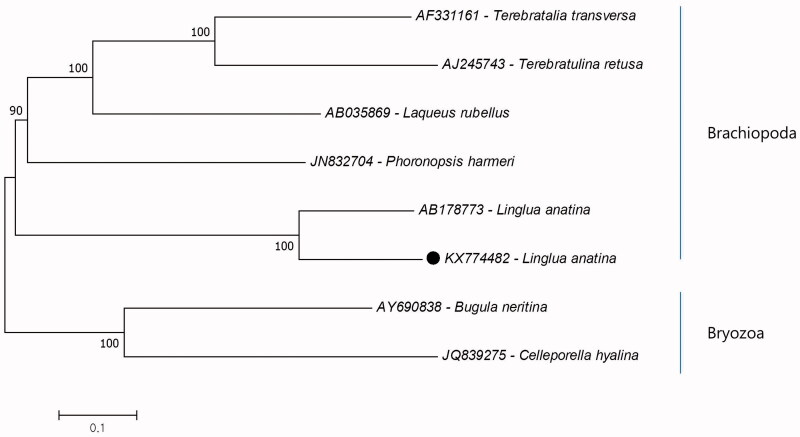
Molecular phylogeny of the Korean *Lingula anatina* in the phylum Brachiopoda. The two outgroup species were chosen from the phylum Bryozoa. The phylogenetic tree was reconstructed by using protein-coding genes of complete mitochondria. The complete mitochondrial genome data retrieved from GenBank. Korean and *L. anatina* marked with black dot.

## References

[CIT0001] Cohen BL, Gawthrop A, Cavalier-Smith T. 1998 Molecular phylogeny of brachiopods and phoronids based on nuclear-encoded small subunit ribosomal RNA gene sequences. Phil Trans R Soc Lond B. 353:2039–2061.

[CIT0002] Endo K, Noguchi Y, Ueshima R, Jacobs TH. 2005 Novel repetitive structures, deviant protein-encoding sequences and unidentified ORFs in the mitochondrial genome of the brachiopod *Lingula anatina*. Mol Evol. 61:36–53. 10.1007/s00239-004-0214-5 15980959

[CIT0004] Karagozlu MZ, Sung JM, Lee JH, Kwon T, Kim CB. 2016 Complete mitochondrial genome sequences and phylogenetic relationship of *Elysia ornata* (Swainson, 1840) (Mollusca, Gastropoda, Heterobranchia, Sacoglossa). Mitochondrial DNA Part B: Resourc. 1:230–232. 10.1080/23802359.2016.1155427 PMC7800206 33473462

[CIT0005] Kim SG, Karagozlu MZ, Kim CH. 2017 Phylogenetic investigations of Lingula anatina among some northwestern Pacific populations, based on mitochondrial DNA cytochrome c oxidase subunit I gene. J Asia-Pacific Biodiversity. 10:162–166.

[CIT0006] Reyment RA, Endo K, Tsujimoto Y. 2007 A note on heterogeneity in northern Pacific populations of the brachiopod species *Lingula an*atina Lamarck. Earth Evol Sci. 1:33–36.

[CIT0007] Saito M, Kojima S, Endo K. 2000 Mitochondrial COI sequences of brachiopods: genetic code shared with protostomes and limits of utility for phylogenetic reconstruction. Mol Phylogenet Evol. 15:331–344. 10860643 10.1006/mpev.2000.0773

[CIT0008] Yang S, Lai X, Sheng G, Wang S. 2013 Deep genetic divergence within a “living fossil” brachiopod *Lingula anatina*. J Paleontol. 87:902–908.

